# Prenatally Diagnosed Retroperitoneal Fetus-In-Fetu with Ipsilateral Testicular Atrophy: A Case Report 

**Published:** 2012-07-01

**Authors:** Shalini Sinha, Yogesh Kumar Sarin, Nita Khurana

**Affiliations:** Department of Pediatric Surgery, Maulana Azad Medical College, New Delhi-110002.; 1 Department of Pathology, Maulana Azad Medical College, New Delhi-110002.

**Keywords:** Fetus-in-fetu, parasitic twin, testicular atrophy, teratoma

## Abstract

We report a case of prenatally diagnosed Fetus-in-fetu (FIF) residing in the left retro-peritoneum in a 2-week-old neonate which was also associated with ipsilateral testicular atrophy. A comparison of features differentiating FIF from a retroperitoneal teratoma, and various theories of origin of FIF are described. The causal relationship of ipsilateral atrophic testis with FIF in this case is also discussed.

## INTRODUCTION

Fetus-in-fetu (FIF) is a parasitic monozygotic monochorionic diamniotic twin that implants itself and grows within the body of its normal karyotypically identical sibling [1]. It is a rare anomaly (around 200 published cases in English literature [2]), which can be differentiated from a retroperitoneal teratoma by the presence of a calcified vertebral axis and limb buds within the mass.

We report a case of prenatally diagnosed FIF residing in the left retroperitoneum and associated with ipsilateral testicular atrophy. Possible causative embryological events are also discussed.

## CASE REPORT

An asymptomatic 2-week-old male neonate was brought to us with an antenatal diagnosis of intra-abdominal cystic mass. He was born at term to a 25-year-old primigravida mother by caesarean section in another hospital, indications being cephalo-pelvic disproportion and fetal distress. It was a non-consanguineous marriage and there was no family history of twins. The new-born weighed 2.2 Kgs and had normal Apgar scores. Antenatal ultrasonography done during the third trimester had shown a spherical fluid-filled cystic mass with 42 mm diameter and 38ml volume having internal ellipsoid calcific and soft tissue areas. The possibility of FIF was considered.

Clinical examination revealed a cystic spherical mass, 4 cm in diameter, in the left hypo-chondrium and lumbar regions. The left testis could not be palpated in the hemiscrotum or inguinal region. Postnatal ultrasound of the abdomen done on day 11 of life demonstrated a thick walled cystic sac with multiple internal ossified contents in the left hypochondrium and lumbar regions. Plain X-Rays of the abdomen were non-contributory. Serum AFP and βhCG were within normal range for age. He underwent exploratory laparotomy through a left upper transverse incision on day16 of life. There was a left sided retroperitoneal cystic mass which was excised after reflecting the left colon medially. No definite vascular pedicle or sac was identified. Postoperative X-ray of the excised specimen showed organized axial skeletal elements (Fig 1). Chromosomal analysis was not performed. Gross examination of the specimen revealed a single globular soft tissue mass measuring 3 x 2 x 2cms (fig 2). The external wall was well encapsulated and had a corrugated skin surface. Sections from the above showed epidermis with appendages. The underlying tissues showed a primitive limb like area with cartilagenous tissue and enchondrial ossification. It was surrounded by skeletal muscle bundles. Interconnecting areas of hyaline cartilage which gave the appearance of vertebral bodies were also found (Fig 3). The above features were consistent with a diagnosis of FIF.

**Figure:**
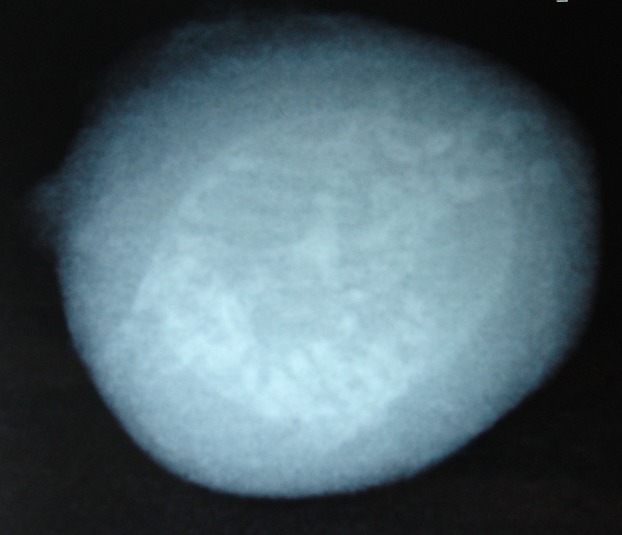
Figure 1: Plain radiograph of the specimen showing vertebral axis as well as long bones.

**Figure:**
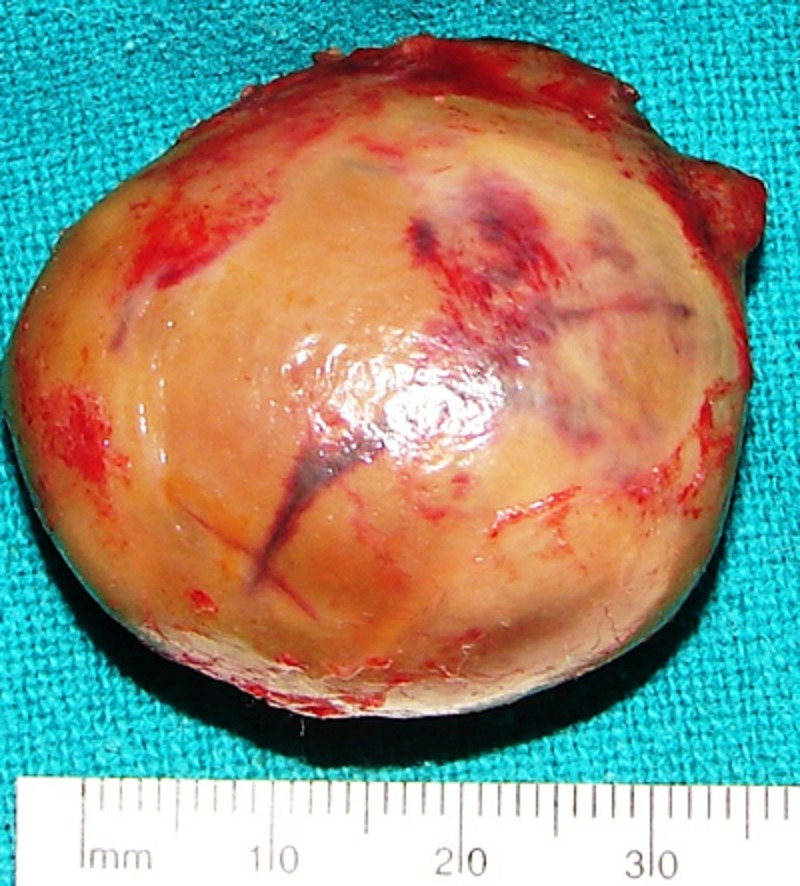
Figure 2: Gross specimen showing well encapsulated skin lined thick wall.

**Figure:**
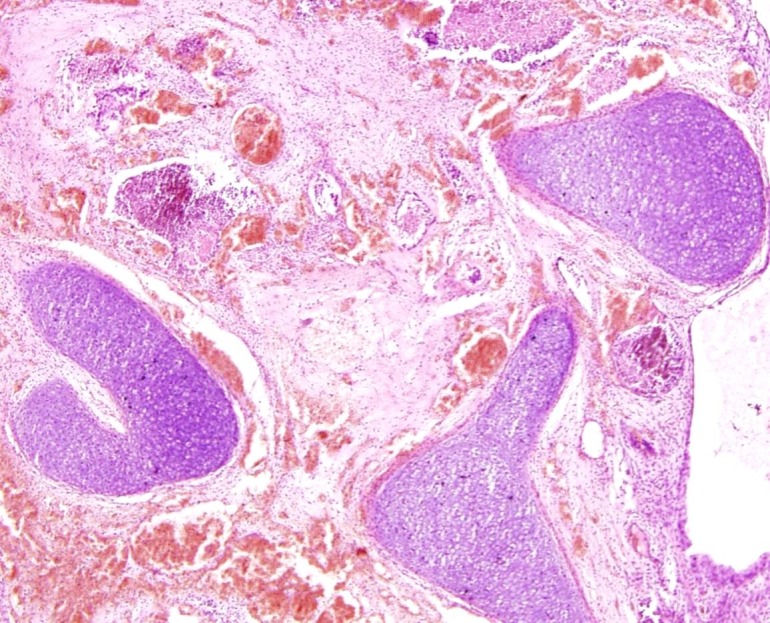
Figure 3: Histopathology section (H and E, Low power field) from the spinal area showing multiple cartilaginous vertebrae.

He was readmitted at the age of 6 months for definitive management of left sided non palpable undescended testis. Pre-operative ultrasound had not revealed any testis in left hemiscrotum or inguinal region. At laparoscopy, the vas deferens was seen entering the left deep ring; however testicular vessels were not visualized. Left inguinal exploration revealed a nubbin representing left atrophic testis that was excised. The parents did not opt for placement of testicular prosthesis at that time. The child has been followed up for 2.5 years and is thriving well; with no features of recurrence on serial abdominal ultrasonography and serum AFP is within normal limits. 

## DISCUSSION

FIF was defined by Willis in 1935 as “a mass containing a vertebral axis often associated with other organs or limbs around this axis” [1]. Since then, the presence of a vertebral axis in a fetiform mass has been considered pathognomonic of FIF. It must be differentiated from a retroperitoneal mature teratoma (RMT) which has an accumulation of pluripotential cells without organogenesis or vertebral segmentation [1]. Some authors consider the two entities as part of a spectrum that includes conjoined symmetric twins; fetus-in-fetu; embryonic vestigial inclusion; and fetiform teratoma [3].

Demography: The incidence of FIF is 1: 500 000 live births with a slight male preponderance [4]. Besides the retroperitoneum which is the most common site of occurrence, it has also been described in the cranial cavity, oral cavity, mediastinum, lung, sacrococcygeal region, kidneys, intra-abdominal and scrotum [4-6].

Clinical presentation and diagnosis: The clinical presentation is either an incidental mass or with symptoms due to compression of adjoining structures in the abdominal, thoracic or cranial cavities [4].

Demonstration of metameric segmentation of its spinal axis on imaging modalities usually clinches the diagnosis. However, non-visualization of the vertebral axis on plain film of the abdomen or on CT scan does not exclude FIF, as the non-calcified vertebrae may be radiolucent, but will definitely be demonstrated on histopathology [4].

Prenatal diagnosis of FIF is possible in about 15% of reported cases (as in our case) [5]. Features suggestive of FIF on imaging have been described as early as 16 weeks of gestation [7]. Prenatal MRI can also be useful. Serum markers like AFP and βhCG may be normal or marginally elevated [8, 9]. The most important differential diagnoses to be considered are teratoma and meconium pseudocyst; both of which will also show calcifications on plain radiography [4]. Besides the above mentioned conditions, intussusception, Wilms’ tumour, neuroblastoma and retroperitoneal lymphadenopathy should also be excluded [10].

Difference between FIF and RMT: Table 1 delineates the various differences between FIF and RMT. A basic point to remember is that unlike teratoma, FIF is not a true tumour.

**Figure:**
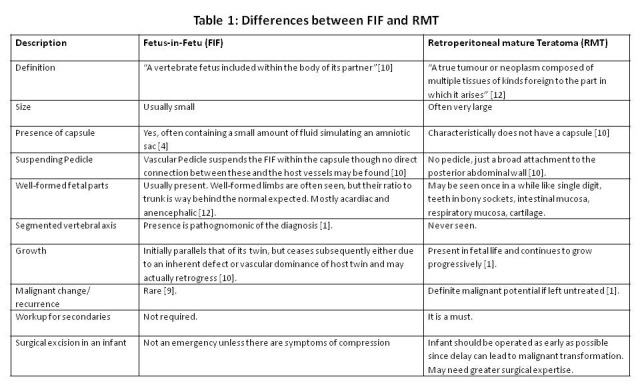
Table 1: Differences between FIF and RMT

Pathology: Detailed and meticulous gross as well as histopathological examination of the excised specimen is the gold standard for diagnosing FIF. The different organs seen in order of frequency are vertebral column (91%), limbs (82.5%), CNS (55.8%), gastrointestinal tract (45%), vessels (40%), and genitourinary tract (26.5%). Hence the vertebral axis may be absent in as much as 9% of cases [4].

Treatment and follow up: Complete surgical excision is the treatment of choice as malignant recurrence has been reported when the membrane was left behind [9]. For the same reasons, these patients should be kept under close follow up with serial ultrasounds and serum tumour markers. Good surgical acumen is needed as injury to the bile duct has also been described during excision of FIF [11].

**Theories of origin**

Included twin theory: This is the most commonly accepted theory of origin of FIF. The supporters of this theory regard FIF as a rare form of monozygotic twinning whereby an aberrant asymmetric twin becomes internalized in the other twin thus acting endoparasitically. Developing from a single ovum, they usually share the same sex, blood group and exactly the same and normal karyotype [4]. This is further strengthened by demonstration of trisomy 21 in both the host as well as FIF by Lee et al [13].

Supporting this school of thought are Spencer's observations that FIF and teratomas are both part of a spectrum of defective conjoint twinning. Both may coexist and there may be multiple foetuses in FIF. He proposed a primary cardiac malformation in the aetiogenesis of FIF with secondary anencephaly [3].

Beaudoin's [6] theory of defective implantation during the second week of development resulting in the invasion of a second embryo (that becomes a homunculus) into the extra-embryonic mesenchyme of the host foetus or autosite, instead of the uterine wall, appears plausible.

The absence of normal umbilical vessels and a definite vascular connection explain the growth retardation and arrest of organ differentiation in almost all cases of FIF [1].

The presence of an axial skeleton implies development of the included fetus past the primitive streak stage when the notochord is formed, a stage thought to be too developed for formation of a teratoma [4].

Fetiform teratoma theory: Willis nurtured the theory that teratomas were derived from embryonic pleuripotential cells associated with the primitive streak, which escaped organizer influence to form a true neoplasm, which may later exhibit benign or malignant characteristics [1]. However, concomitant presence of FIF with mature or immature teratoma in several instances implies a common embryological origin [14]. Supporters of this theory believe that FIF represents a well- differentiated, highly-organized or fetiform teratoma [8].

Association of FIF with ipsilateral atrophic testis: The normal descent of testis is known to occur during late gestation. Any retroperitoneal mass lesion during fetal life can hinder the descent or vascularity of the testis resulting in cryptorchidism or anorchia respectively. Since the FIF in our case was small it affected only the ipsilateral testis. The testicular atrophy could have resulted from extrinsic compression of the testicular vessels by the FIF, since the testicular vessels could not be identified during laparoscopy. Moreover, the FIF may have been larger to begin with producing pressure effects, and regressed later to its small size. Pelvic or large retroperitoneal FIF have been reported to cause bilateral testicular maldescent [15]. However, to the best of our knowledge, testicular atrophy associated with retroperitoneal FIF has not been reported till date in English literature.

To conclude, with improvement in imaging modalities, more cases of FIF are being diagnosed preoperatively and even prenatally (as in our case). The association of FIF with ipsilateral testicular atrophy was also noteworthy. 

## Footnotes

**Source of Support:** Nil

**Conflict of Interest:** None

